# Screening of metabolic modulators identifies new strategies to target metabolic reprogramming in melanoma

**DOI:** 10.1038/s41598-021-83796-8

**Published:** 2021-02-23

**Authors:** Cecilie Abildgaard, Salvatore Rizza, Helle Christiansen, Steffen Schmidt, Christina Dahl, Ahmad Abdul-Al, Annette Christensen, Giuseppe Filomeni, Per Guldberg

**Affiliations:** 1 Molecular Diagnostics Group, Danish Cancer Society Research Center, Strandboulevarden 49, 2100 Copenhagen, Denmark; 2 Department of Clinical Genetics, Lillebaelt Hospital - University Hospital of Southern Denmark, Vejle, Denmark; 3 Redox Biology Group, Danish Cancer Society Research Center, Copenhagen, Denmark; 4 Lundbeckfonden Center of Excellence NanoCAN, Institute of Molecular Medicine, University of Southern Denmark, Odense, Denmark; 5 Molecular Oncology, Institute of Molecular Medicine, University of Southern Denmark, Odense, Denmark; 6 Department of Biology, Tor Vergata University of Rome, Rome, Italy; 7 Center for Healthy Aging, Copenhagen University, Copenhagen, Denmark; 8 Department of Cancer and Inflammation Research, Institute for Molecular Medicine, University of Southern Denmark, Odense, Denmark; 9 Present Address: Roche Innovation Center Copenhagen, Hørsholm, Denmark

**Keywords:** Melanoma, Cancer, Cancer therapy, Drug discovery

## Abstract

The prognosis of metastatic melanoma remains poor due to de novo or acquired resistance to immune and targeted therapies. Previous studies have shown that melanoma cells have perturbed metabolism and that cellular metabolic pathways represent potential therapeutic targets. To support the discovery of new drug candidates for melanoma, we examined 180 metabolic modulators, including phytochemicals and anti-diabetic compounds, for their growth-inhibitory activities against melanoma cells, alone and in combination with the BRAF inhibitor vemurafenib. Two positive hits from this screen, 4-methylumbelliferone (4-MU) and ursolic acid (UA), were subjected to validation and further characterization. Metabolic analysis showed that 4-MU affected cellular metabolism through inhibition of glycolysis and enhanced the effect of vemurafenib to reduce the growth of melanoma cells. In contrast, UA reduced mitochondrial respiration, accompanied by an increase in the glycolytic rate. This metabolic switch potentiated the growth-inhibitory effect of the pyruvate dehydrogenase kinase inhibitor dichloroacetate. Both drug combinations led to increased production of reactive oxygen species, suggesting the involvement of oxidative stress in the cellular response. These results support the potential use of metabolic modulators for combination therapies in cancer and may encourage preclinical validation and clinical testing of such treatment strategies in patients with metastatic melanoma.

## Introduction

Nearly 300,000 new cases of cutaneous melanoma are reported each year worldwide, with a high increase in incidence among young people^1^. The prognosis of metastatic melanoma has historically been poor with a 3-year survival rate of ~ 15% and a median survival of < 12 months, which can be ascribed to the aggressive nature of the disease and low response rates to conventional chemotherapy^2^. In recent years, major therapeutic advances have been made, including the development of novel targeted therapies such as small-molecule inhibitors targeting the MAPK pathway, which is consistently dysregulated in melanoma. Several of these drugs have been approved as first-line treatment for advanced melanoma, including inhibitors of MEK and BRAF, which can cause a rapid decrease in tumor burden^3^. Despite high initial response rates, acquired resistance to these drugs is common, and most patients experience relapse within months. More recently, longer-lasting responses have been achieved with immune checkpoint inhibitors (ICIs), which activate the host’s antitumor immune response. However, > 75% of patients have limited benefit from this treatment, and the side effects are often severe^3^. Thus, although the introduction of MAPK inhibitors and ICIs in the clinic has led to a decrease in melanoma mortality, new treatment modalities are needed to improve outcomes.

Melanoma arises from melanocytes, the cells producing and distributing pigment in the skin and eyes. The transformation from the quiescent state of melanocytes to the highly proliferative and invasive phenotype of melanoma cells alters the demand for energy and building blocks and requires reprogramming of cellular metabolism. The malignant state is associated with higher glycolytic activity and utilization of glucose, as well as lower mitochondrial respiration even under normoxic conditions^4^. This metabolic profile is known as the Warburg effect and is a hallmark of many cancer types^5^. The most prevalent *BRAF* mutation, p.V600E, is directly implicated in cellular metabolic reprogramming by inducing hyperactivation of the MAPK pathway and promoting the enrichment of glycolytic enzymes^6,7^. Treatment with BRAF inhibitors reverses the Warburg effect by reducing glycolytic activity and stimulating mitochondrial biogenesis through the MITF-PGC1α pathway^8,9^. This metabolic shift highlights the flexibility of melanoma cells to adapt to new metabolic demands.

Given the frequent development of resistance to targeted melanoma monotherapies, it is important to identify drug combinations that can lead to durable patient responses. One such strategy is to co-target components of the MAPK pathway, such as BRAF and MEK, which has proven to be effective in the treatment of metastatic melanoma^10–12^. However, as with single-agent therapies, the initial effect diminishes over time due to acquired resistance, eventually leading to relapse^13,14^. Targeting metabolic pathways together with the use of oncogene inhibitors has been suggested as an alternative strategy to overcome resistance and improve therapeutic efficacy^15–17^.

Some metabolic modulators, including the pyruvate dehydrogenase kinase inhibitor dichloroacetate (DCA) and the biguanide metformin, sensitize melanoma cells to BRAF inhibitors^18,19^. Accumulation of reactive oxygen species (ROS) and reduced energy production have been suggested as mechanisms responsible for this sensitization to BRAF inhibition^18,20,21^. Other metabolic modifiers can be potentially relevant for melanoma, including drugs previously used as anti-diabetics as well as phytochemicals. Many anti-diabetic compounds are pharmacologically well characterized and have been tested in clinical trials for diabetes. The anti-diabetic effects are often attributed to systemic effects, but many of these drugs have molecular targets involved in the regulation of cellular metabolism^22,23^. Phytochemicals encompass a class of bioactive compounds naturally occurring in plants and foods, some of which have been used for preventive or therapeutic purposes in traditional medicine. Phytochemicals are also exploited in modern medicine, where their mechanisms of action might involve modulation of cellular metabolism^24,25^. For some of these compounds, synthetic derivatives have been developed to improve potency and specificity^20,26^.

In this study, we sought to identify metabolic modifiers exerting a growth-inhibitory effect on melanoma cells. Given the metabolism-modifying effects of targeted BRAF inhibition, we specifically investigated the potential of combination treatments. Hence, we designed a screen to examine the effects of 180 metabolic modifiers, alone and in combination with the clinically approved BRAF inhibitor vemurafenib. Two positive hits from this screen, 4-methylumbelliferone (4-MU) and ursolic acid (UA), were selected for further investigation and metabolic characterization.

## Results

### Screen-based identification of metabolic modulators reducing the viability of melanoma cells

A library of 180 metabolic modulators was screened for their effects on cellular viability of BRAF^V600E^-mutated melanoma cells (ED-013 and ED-196) in the presence and absence of vemurafenib. Inherently vemurafenib-resistant melanoma cells harboring the NRAS^Q61K^ mutation (ED-094) and melanoma cells with acquired resistance to vemurafenib (ED-013-R2) were included as controls in the screen.

Before determining the screening concentration for vemurafenib, IC_50_ values were determined using an experimental set-up corresponding to the screening conditions (Supplementary Fig. S1). All cell lines were treated with vemurafenib at various concentrations, and IC_50_ values were determined as the reduction in relative viability using the CellTiter-Blue assay (Table 1). The IC_50_ value for ED-196 cells (330 nM) was in accordance with a previous study^27^. As expected, ED-013 and ED-196 cells were sensitive to vemurafenib, and vemurafenib resistance was confirmed in ED-013-R and ED-094 cells (Supplementary Fig. S2). Based on the IC_50_ values of the sensitive cell lines, 0.5 µM was chosen as the final concentration for the screen. All remaining compounds were tested at a concentration of 10 µM, which is within the range typically used in high-throughput screening^28–30^. Using an automated setup, cells were seeded according to the protocol outlined in Supplementary Fig. S1 and treated in triplicate with each screening compound for 5 days, alone and in combination with vemurafenib.

**Table 1:** IC_50_ values.

	4-MU (µM)	UA (µM)	Vemurafenib (µM)	DCA (mM)*
ED-013	472 ± 17	11.8 ± 1.7	0.48 ± 0.16	14.4 ± 2.0
ED-196	529 ± 80	13.2 ± 2.0	0.33 ± 0.12	35.8 ± 3.2

*Values reported by Abildgaard et al.^18^.

The criteria for selecting positive hits were: *i*) an enhancing effect on vemurafenib cytotoxicity (reducing the viability with at least 10% more than vemurafenib alone) and *ii*) a low cytotoxic effect alone (< 10% reduction in viability). The compounds tested were ranked according to the magnitude of the difference between the relative viability with and without vemurafenib, incorporating the results for both BRAF^V600E^-mutated cell lines (Supplementary Table S1). The average of the differences plus the standard deviation was selected as the cutoff value. Positive hits were selected if they met the cutoff in both ED-013 and ED-196 cells. Based on these criteria, 4-MU and UA were identified as the top positive hits.

### 4-MU and UA inhibit melanoma growth

In the screen outlined above, relative cellular viability was determined as a measure of metabolic activity. To assess the effects of 4-MU and UA on cell growth more directly, ED-013 and ED-196 cells were exposed to a range of concentrations for 6 days, and cell viability was evaluated using a crystal violet assay. The resulting dose–response curves (Supplementary Fig. S3) showed that the two cell lines responded similarly to each compound, with no significant difference between their IC_50_ values (Table 1). At low concentrations (≤ 10 µM), 4-MU slightly increased cell viability in both cell lines (Supplementary Fig. S3); however, this increase was not observed in the presence of 0.1 µM vemurafenib (data not shown). At higher concentrations of 4-MU (≥ 500 μM), cell viability was markedly reduced (Supplementary Fig. S3). Treatment with UA led to a steep reduction in cell viability from ~ 80% to < 20% at concentrations between 10 and 20 µM (Supplementary Fig. S3).

### 4-MU and UA differently affect cellular metabolism

**Figure 1:**
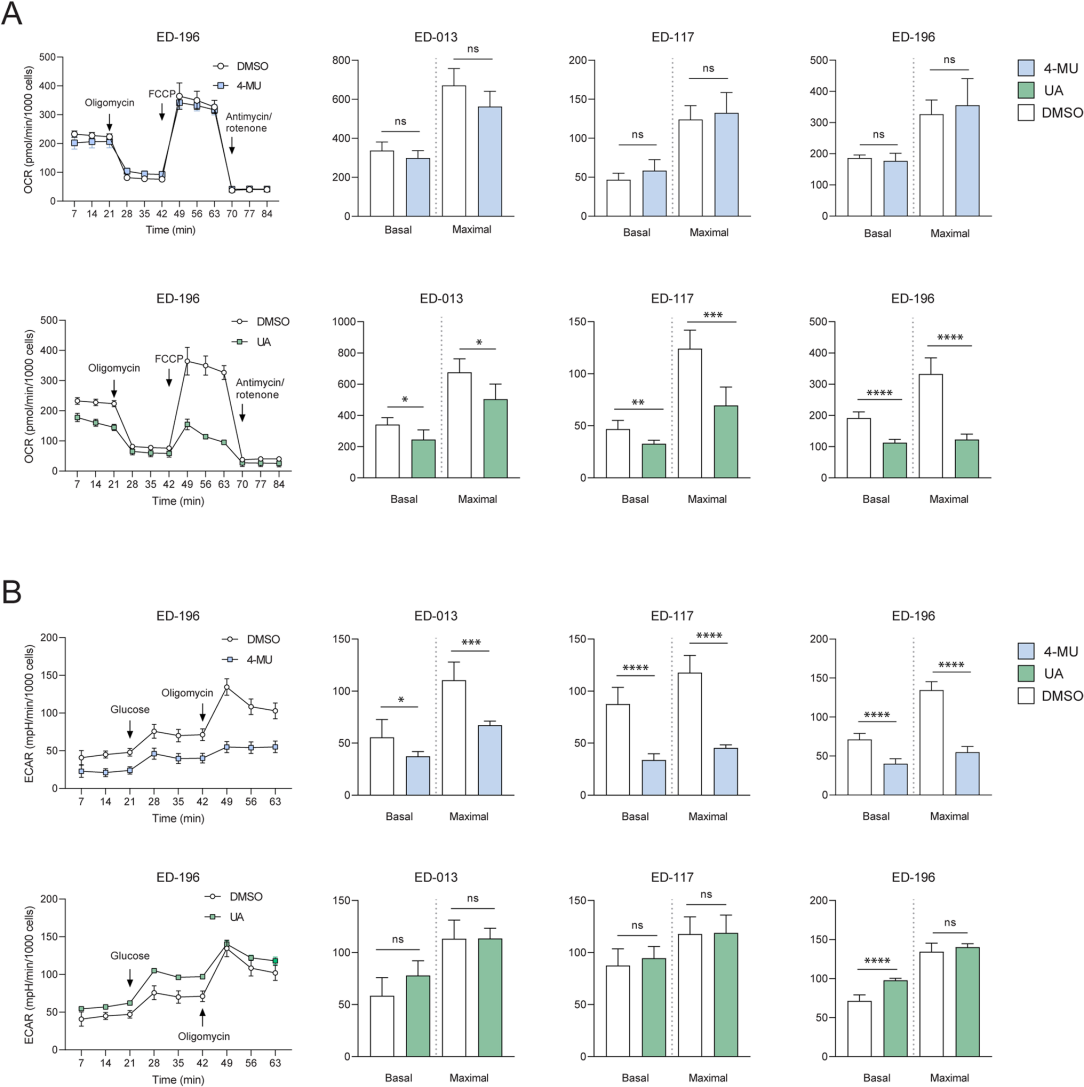
Effects of 4-MU and UA on cellular metabolism. Mitochondrial (**A**) and glycolytic (**B**) profiles of ED-013, ED-117 and ED-196 cells treated with 4-MU (100 μM; upper panel) or UA (10 μM; lower panel) for 24 h compared to untreated controls. The data points represent the mean ± SD of 6 parallel Seahorse XFe96 measurements of OCR (**A**) and ECAR (**B**) during successive injection of glucose (10 mM), oligomycin (2 μM), FCCP (1 μM) and rotenone/antimycin (1 μM/1 μM). (*p < 0.05; ** *p* < 0.01; *** *p* < 0.001; **** *p* < 0.0001; ns—not significant).

To assess the effects of 4-MU and UA on melanoma cell metabolism, we evaluated OCR (a measure of mitochondrial respiration) and ECAR (a parameter related to glycolytic efficiency) using the Seahorse XFe96 Analyzer. In addition to the two cell lines used in the screen, the BRAF^V600E^-mutated melanoma cell line ED-117 was included as a biological replicate. All cell lines were treated with 4-MU (100 µM) or UA (10 µM) for 24 h. 4-MU did not significantly affect basal respiration or respiratory capacity in either cell line (Fig. 3A). In contrast, UA induced a reduction in basal respiration in all cell lines and a collapse of the respiratory capacity in ED-196, keeping respiration close to the basal level even upon stimulation with FCCP (Fig. 1A). In 4-MU treated cells, a marked drop in both basal glycolytic activity and capacity was observed (Fig. 1B). In UA-treated ED-196 cells, the reduced respiration correlated with an increase in basal glycolysis, but not in glycolytic capacity. This suggested that 4-MU and UA exerted opposing effects on cellular metabolism, with 4-MU acting as a glycolytic inhibitor and UA impairing mitochondrial respiration. The increase in basal glycolytic activity induced by UA might be interpreted as a compensatory mechanism to sustain energy production.

### 4-MU potentiates the effect of vemurafenib

**Figure 2:**
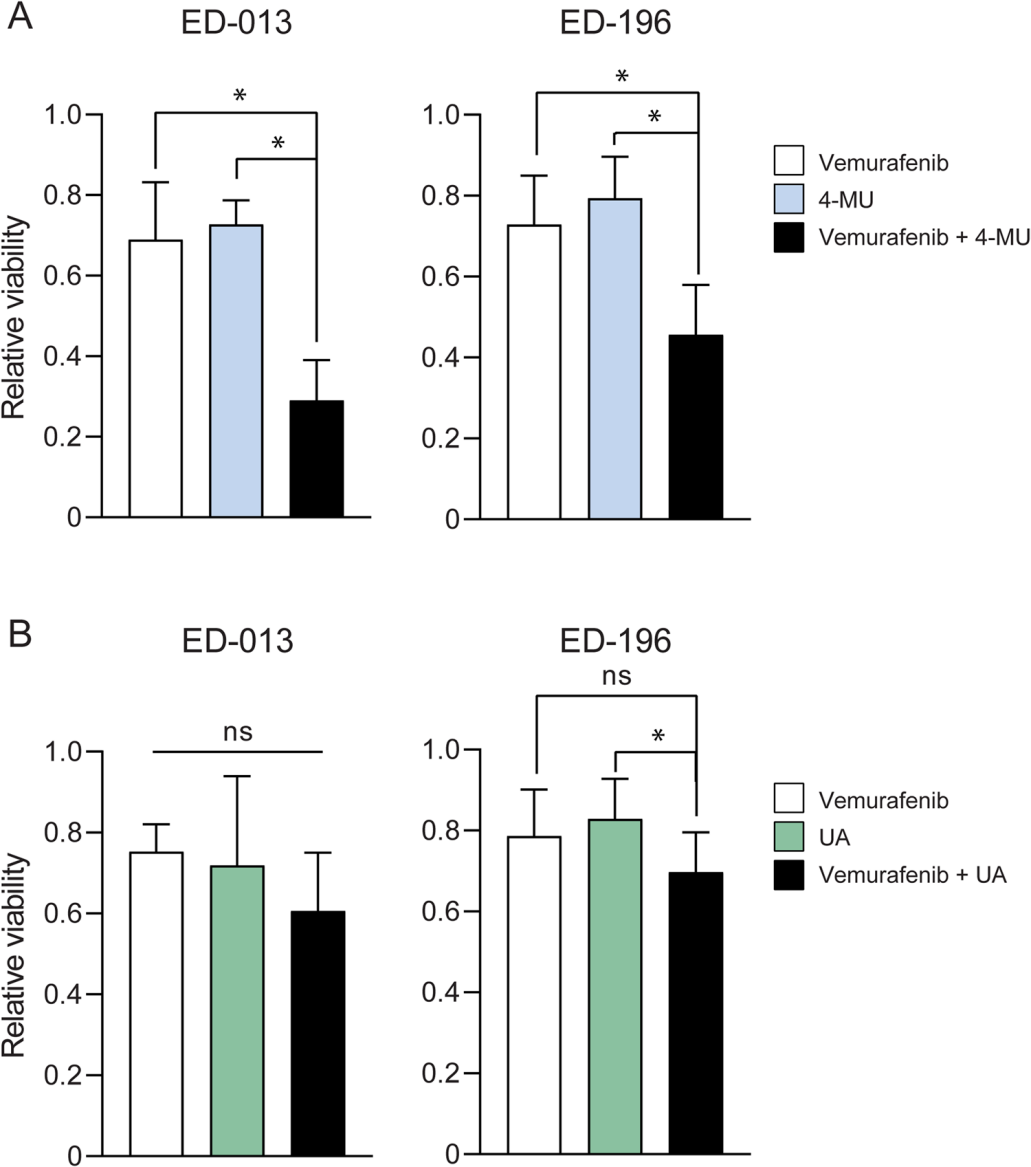
Effects on melanoma cell viability of 4-MU and UA in combination with vemurafenib. Relative viability of ED-013 and ED-196 cells was determined with crystal violet staining after treatment with (**A**) 4-MU (400 µM), vemurafenib (0.1 µM) and the combination, or (**B**) UA (10 µM), vemurafenib (0.1 µM) and the combination for 6 days. A one-way ANOVA was performed to determine variance between the treatment groups. Data represent the mean ± SD of 3 individual experiments. Tukey’s HSD test was performed to determine statistical significance (*p < 0.05; ns—not significant).

To validate the screening results, ED-013 and ED-196 cells were treated with 4-MU (400 µM) and UA (10 µM) alone and in combination with vemurafenib (0.1 µM) for 6 days, and the relative cell viability was determined using the crystal violet assay. A lower concentration of vemurafenib than the one used in the screen was chosen because both cell lines showed a higher sensitivity to vemurafenib when using the crystal violet assay compared with the CellTiter-Blue assay (data not shown). The results obtained in these experiments showed that the combination of 4-MU and vemurafenib was more effective in reducing the viability of both cell lines than either compound alone (Fig. 2A). In contrast, UA did not potentiate the effect of vemurafenib across a broad range of concentrations (Fig. 2B and Supplementary Fig. S4).

### UA enhances the effect of DCA on melanoma growth

**Figure 3:**
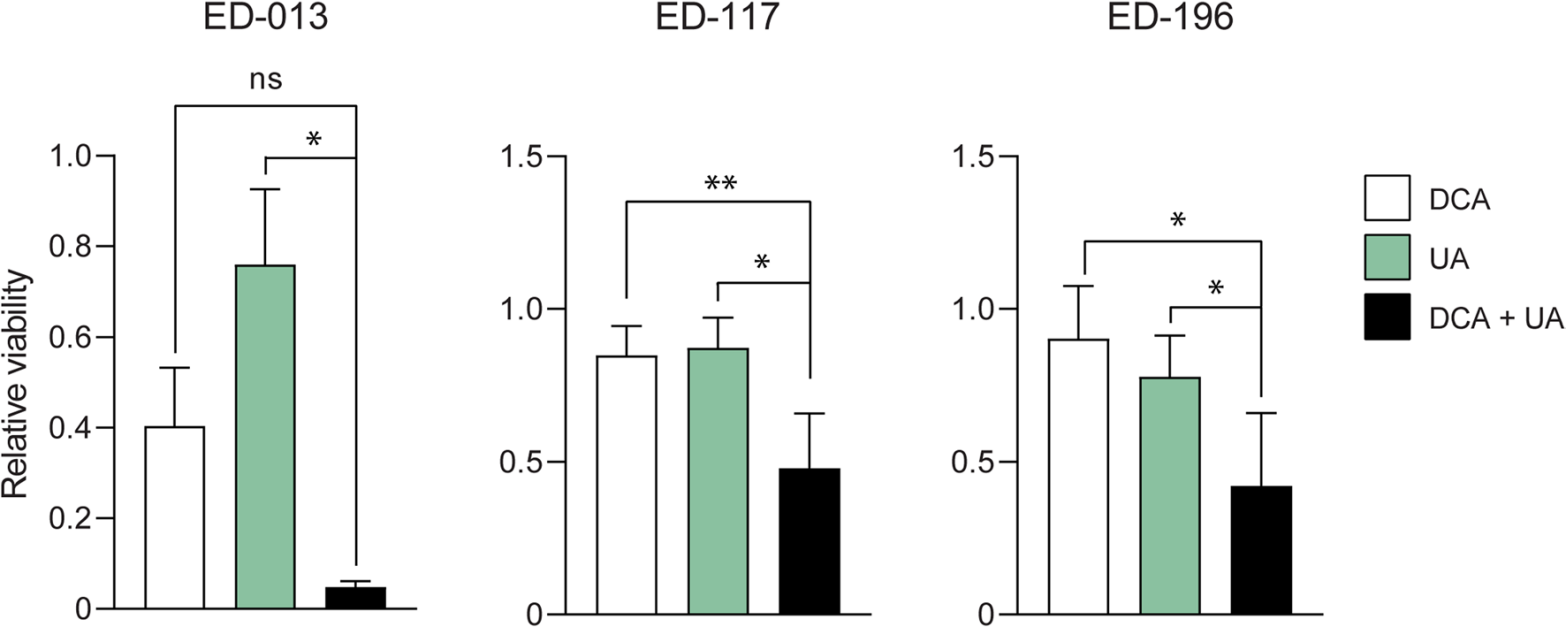
Effects of UA and DCA on melanoma cell viability. Relative viability of ED-013, ED-117 and ED-196 cells was determined with crystal violet staining after treatment with UA (10 µM), DCA (10 mM) and the combination for 6 days. A one-way ANOVA was performed to determine variance between the treatment groups. Data represent average values ± SD of 3 individual experiments. Tukey’s HSD test was performed to determine statistical significance (*p < 0.05; ** *p* < 0.01; ns—not significant).

It was previously shown that compounds promoting a metabolic switch towards higher glycolytic dependence synergizes with DCA to reduce the growth of melanoma cells^27^. Since UA was shown to promote an increase in glycolytic activity (Fig. 1B), we hypothesized a more robust anti-melanoma effect of UA if used in combination with DCA. We, therefore, treated melanoma cells with UA (10 µM) and DCA (10 mM), as single agents and in combination, and determined the relative viability using the crystal violet assay. The results displayed in Fig. 3 demonstrated a stronger growth-reducing effect of UA and DCA in combination than either compound alone, for all three cell lines.

### 4-MU and UA induce ROS production in combination with vemurafenib and DCA, respectively

**Figure 4:**
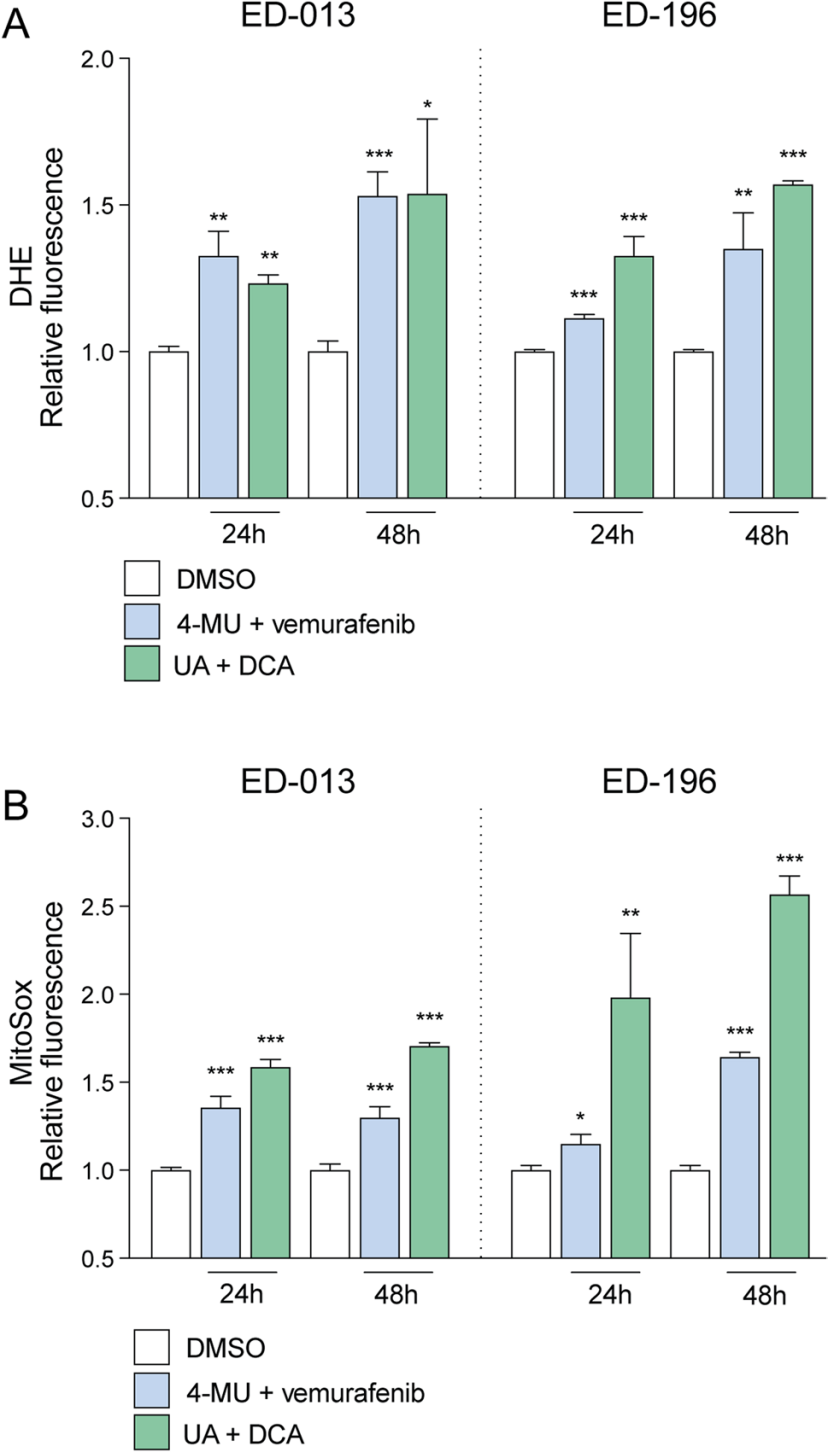
Effects of 4-MU + vemurafenib and UA + DCA on the production of superoxide. Cytofluorimetric determination of cellular superoxide *p* < **A**) and mitochondrial superoxide (**B**) in ED-013 and ED-196 melanoma cells upon incubation with the probes DHE and MitoSOX, respectively. Cells were treated with 4-MU (100 µM) in combination with vemurafenib (0.1 µM), or with UA (10 µM) in combination with DCA (10 mM) for 24 or 48 h. Values are reported as relative fluorescence in treated cells compared to vehicle only (DMSO)-treated cells. Data represent the mean ± SEM of 3 independent experiments done in triplicate. An unpaired t-test was performed to determine statistical significance (*p < 0.05; ** *p* < 0.01, *** *p* < 0.001).

Metabolism defects are commonly associated with increased ROS production and massive oxidative damage. This condition is induced by several classes of chemotherapeutics, mainly those exerting cytotoxicity by affecting metabolic pathways required to obtain ATP and reducing power^31,32^. On the basis of this consideration, we aimed at evaluating ROS (i.e. superoxide anion) in cells exposed to UA + DCA or 4-MU + vemurafenib by cytofluorometric analyses performed with two different probes, i.e. DHE (estimating total superoxide produced in the cells) and MitoSOX (specifically detecting superoxide generated in the mitochondria). Results obtained in ED-013 and ED-196 cells indicated that both treatment combinations were associated with a time-dependent increase in the cellular concentrations of superoxide (Fig. 4A). However, MitoSOX fluorescence differed significantly between the two treatments, with UA + DCA being associated with higher levels of mitochondrial superoxide than those produced by 4-MU + vemurafenib (Fig. 4B). This observation confirmed previous metabolic analyses of OCR and ECAR and argued for the combination of UA and DCA being selectively detrimental to mitochondria.

### Drug combinations differently affect cell growth and viability

**Figure 5:**
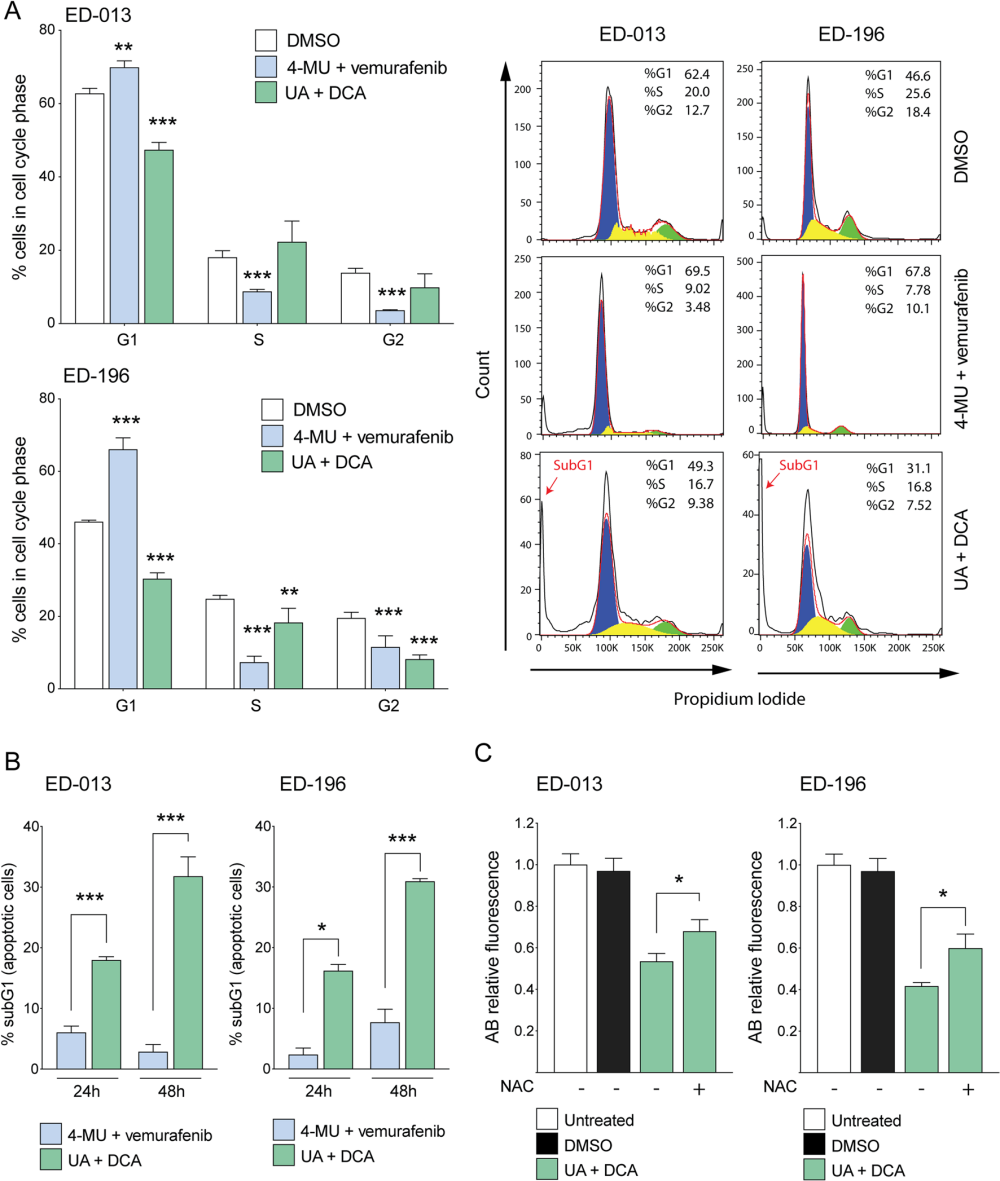
Cytostatic and cytotoxic effects of 4-MU + vemurafenib and UA + DCA in melanoma cells. (**A**) Cytofluorimetric evaluation of cell cycle in ED-013 and ED-196 melanoma cells upon incubation with propidium iodide (PI) upon treatment with 4-MU (100 µM) + vemurafenib (0.1 µM), or UA (10 µM) + DCA (10 mM) for 48 h. Percent of cells in the different phases of cell cycle was determined by FlowJo v 10.1 software. Data show the mean of 3 independent experiments ± SD. A two-way ANOVA was performed to determine statistical significance (**p < 0.01, *** *p* < 0.001). Representative plots are shown on the right. (**B**) Percentage of apoptotic cells in culures of ED-013 and ED-196 treated with 4-MU + vemurafenib or UA + CA for 24 or 48 h. The hypodiploid nuclei (subG1) population was gated by flow cytometry upon staining with PI. Data represent the mean ± SD of 3 independent experiments. An unpaired t-test was performed to determine statistical significance (*p < 0.05; *** *p* < 0.001). (**C**) Analysis of cell viability in the same cell lines upon staining with Alamar Blue (AB) upon 48 h of treatment with UA in combination with DCA in the presence or absence of the antioxidant *N*-acetyl-l-cysteine (NAC, 5 mM). DMSO was used as vehicle. Values are reported as relative fluorescence of AB in treated compared to untreated cells. Data represent the mean ± SD of 3 independent experiments. An unpaired t-test was performed to determine statistical significance (*p < 0.05).

In order to characterize the anti-melanoma activity induced by UA + DCA and 4-MU + vemurafenib, we treated cells for 48 h and then performed cytofluorometric analyses upon propidium iodide staining. The results shown in Fig. 5A indicate that 4-MU + vemurafenib induced cell cycle arrest in G1 phase, whereas UA + DCA triggered a general decrease in the number of cells in each phase analyzed (see representative cytofluorometric histograms on the right). This result was in line with the significant increase of the SubG1 (apoptotic) population observed only in UA + DCA-treated cells (Fig. 5B), and, overall, suggested different modalities through which the two drug combinations affected melanoma growth, with 4-MU + vemurafenib being more cytostatic and UA + DCA compromising cell viability.

To eventually understand if oxidative stress played a driving role in UA + DCA-induced cell death, we pre-incubated the cells with a well-known antioxidant, *N*-acetyl-l-cysteine (NAC), and analyzed cell viability using an Alamar blue assay. NAC partly counteracted cytotoxicity induced by UA + DCA (Fig. 5C). However, crystal violet staining, performed at longer time points (up to 6 days), indicated that NAC was unable to maintain cell survival, even at high doses (20 mM; data not shown). These results suggest that ROS contributed to, but were not the only molecular determinant of, melanoma cell death induced by treatment with a combination of UA and DCA.

To investigate the effect on cellular energetics, we investigated the phosphorylation status of the energy sensor AMPK in ED-013 and ED-196 cells treated with UA + DCA or 4-MU + vemurafenib. Neither drug combination led to the phosphorylation of AMPK, suggesting that the cells were not under energetic stress (Supplementary Fig. S5).

## Discussion

Metabolic modulation has been widely accepted as a strategy to sensitize cancer cells to a range of different therapies or to prevent the development of treatment resistance^33–35^. Here we performed a screen of 180 metabolic modulators for viability-reducing effects in melanoma cells in combination with the BRAF inhibitor vemurafenib. One of the positive hits identified in this screen was 4-MU, a derivative of coumarin that has been approved in Europe and Asia for the treatment of biliary spasm under the name hymecromone. 4-MU inhibits the synthesis of hyaluronic acid (HA) and has demonstrated anti-tumor activity in several cancer cell lines in vitro, with a potency similar to that observed here^36–38^. Our results from metabolic analysis provide evidence that 4-MU inhibits glycolysis at a concentration below the IC_50_. This is in agreement with recent results showing that 4-MU-treated chondrocytes have reduced glycolytic rate and that this effect of 4-MU is more important than inhibition of HA synthesis in modifying the phenotype of chondrocytes^39^. From a biochemical point of view, inhibition of glycolysis by 4-MU can be explained in terms of the equilibria that cancer cells should maintain among different metabolic pathways that utilize glucose, namely: (1) HA de novo synthesis, required for the production of extracellular matrix polysaccharides; (2) glycolysis, needed to generate pyruvate and ATP; and (3) the pentose phosphate pathway (PPP), essential to produce ribose-5P (a nucleotide precursor) and NADPH.

In combination experiments, 4-MU was shown to potentiate the effect of vemurafenib on growth inhibition of BRAF^V600E^-mutant melanoma cells. As 4-MU and vemurafenib both suppress glycolytic activity, cells treated with these drugs would become increasingly dependent on mitochondrial respiration for energy production. Considering that mitochondria are often dysfunctional in melanoma cells^6,40^, this metabolic switch could inhibit cell growth through at least two different mechanisms, namely increased production of ROS and energy deficiency. Our data suggest that oxidative stress is the main effector mechanism, as melanoma cells treated with 4-MU and vemurafenib showed increased ROS levels, but not increased activation of the cellular energy stress sensor AMPK. Still, the two mechanisms may not be mutually exclusive, and we cannot exclude that energy deficiency caused by a decrease in ATP production could become a limiting factor under different cellular conditions.

The other positive hit identified in the screen, UA, is a naturally occurring triterpene found in apple peel and in certain other fruits and vegetables and exploited in traditional medicinal herbs. It belongs to a group of terpenoids renown for their anti-inflammatory, anti-proliferative, and pro-apoptotic effects^41,42^. Several studies have revealed that UA also possesses anti-cancer properties^26,43–46^, and recent studies demonstrated reduced proliferation and invasive potential of melanoma cell lines exposed to UA^47–49^. Although the effects of UA on cellular metabolism are still not well defined, several studies indicate that it damages mitochondria, as it induces depolarization and leads to ROS accumulation in different cancer cell types, such as breast cancer^50^, hepatocellular carcinoma and melanoma^26^. These observations are in line with our results, which showed a dramatic reduction of mitochondrial respiration in melanoma cells after treatment with UA, a condition that was associated with an increase in ECAR. This switch might potentially compensate for the reduction in the glycolytic activity induced by vemurafenib in combination treatment and would explain why we did not observe a synergistic effect between these two compounds. Since the CellTiter-Blue assay used in the screen provides a measure of metabolic activity, the observed synergistic effect could be exclusively associated with the metabolic rate and not with cellular growth.

While UA did not potentiate the effect of vemurafenib on melanoma cell growth, it showed high toxicity when used in combination with the PDK inhibitor DCA. Indeed, by accelerating the uptake of pyruvate—the end product of glycolysis—into the mitochondria, DCA maximizes the effects of UA on cell metabolism and enhances its mitochondrial toxicity. In contrast to vemurafenib, DCA does not act against but sustains the high glycolytic rate induced by UA. However, the resulting overload of NADH speeds up the electron flow through the respiratory chain, a condition that, in the presence of UA, enhances mitochondrial depolarization and ROS production. This hypothesis is supported by data obtained in this work, showing increased mitochondrial superoxide production and apoptosis upon combined treatment with UA and DCA. Moreover, it is in line with our previous report suggesting that the anti-melanoma effect of DCA can be amplified by promoting glycolytic dependence through inhibition of RAR-β signaling^27^. The amount of literature supporting the rationale of using DCA in cancer treatment is extensive^51^. Furthermore, several synthetic, structurally modified derivatives of both UA and DCA have been developed, which possess improved potency and cancer specificity^20,26^.

In conclusion, this study has identified two novel strategies to target metabolic reprogramming in melanoma. We demonstrated that switching metabolism away from glycolysis toward mitochondrial respiration with 4-MU enhances the effect of vemurafenib in BRAF^V600E^-mutated melanoma cells, and that promoting a glycolytic shift with UA enhances the effect of DCA. The low cytotoxicity of 4-MU, UA and DCA has been demonstrated in vitro in models of different non-malignant cell types^18,48,52,53^, and the safety of administering these drugs to patients has been extensively documented in clinical trials^54–59^. While the combination of 4-MU and vemurafenib is applicable only to BRAF-mutated melanomas, combined treatment with UA and DCA might in theory be applied more broadly in cancers that rely upon glycolysis to sustain their growth.

## Methods

### Compound library

Two libraries consisting of 143 phytochemicals and 31 anti-diabetic compounds, respectively, were purchased from Selleckchem. Six additional metabolic modulators (lipoic acid, neocuproine, thiamine, DCA, hydroxycitrate, and retinoic acid) were purchased from Sigma-Aldrich and added to the screening library, yielding a total of 180 compounds. The full list of compounds is shown in Supplementary Table S1. The compounds of the two pre-composed libraries were dissolved in DMSO to a concentration of 100 µM. Lipoic acid was dissolved in ethanol, neocuproine in methanol, thiamine, DCA, and hydroxycitrate in water, and retinoic acid in DMSO, all to a concentration of 100 µM. With medium added to the cell culture plates, each compound had a final concentration of 10 µM. Vemurafenib was purchased from Selleckchem and dissolved in DMSO.

### Cell lines

Melanoma cell lines were obtained from the European Searchable Tumour line Database (ESTDAB, ED)^60^. ED-013 (FM-55-M2), ED-196 (Mel-DO) and ED-117 (Mel-NT3-00) all have the BRAF^V600E^ mutation, whereas ED-094 (Mel-LE) has the NRAS^Q61K^ mutation^61^. ED-013-R2 is a vemurafenib-resistant derivative of ED-013^18^. Cell lines were authenticated by testing for unique oncogenic mutations. Cells were cultured at 37 °C under 5% CO_2_ in RPMI-1640 medium supplemented with 10% fetal bovine serum (FBS). ED-013-R2 cells were cultured in the same medium supplemented with 1 µM vemurafenib. All media and supplements were purchased from Invitrogen.

### Pre-screening analysis

#### Seeding optimization

Before high-throughput screening, the automatic seeding procedure was optimized for each cell line. Seeding densities and liquid handling procedures were optimized to provide the lowest variation. Seeding was performed in a 96-well format (Low Edge Effect plates; Thermo Fisher Scientific) using a MultiDrop Combi 96w reagent dispenser (Thermo Fisher Scientific). Different pipetting schemes were tested to determine whether the order of adding the screening compounds and cell suspensions affected drug sensitivity. The selected protocol employs a “reverse addition” setup, where the compounds are added before the cells are seeded (Supplementary Fig. S1). With this setup, most of the surface of the cells is exposed to the compounds and the cell lines showed a higher sensitivity than when adding the compounds after cell seeding (data not shown).

#### Solvent testing

Effects of the solvents (DMSO, water, ethanol, and methanol; 0.07–10%) on cell viability were tested for all cell lines at two time-points (2 and 5 days) and two cell densities (1500 and 2500 cells/well). Only DMSO caused a reduction in cell viability, and only at a concentration above 0.6%. Since the final concentration of any solvent in the screen was 0.1%, the contribution from solvents was considered minimal. All solvent combinations were included on each plate in the screen along with untreated controls.

### Screening of metabolic modulators in combination with vemurafenib

High-throughput screening was performed using a large-scale integrated robotic workstation, provided by Beckman Coulter/Ramcon. ED-013, ED-013-R2, and ED-094 were seeded at a density of 2000 cells/well; ED-196 was seeded at 2500 cells/well due to a lower doubling time. After addition of test compounds and cell seeding, the plates were incubated for 5 days. The end-point measurements of cell viability were performed using a fluorometric CellTiter-Blue assay (Promega). After the addition of the CellTiter-Blue reagent, cells were incubated at 37 °C and 5% CO_2_. The fluorescent signal was measured at 560/590 nm after 1 and 3 h. The short incubation time was included to avoid oversaturation in wells with many viable cells, whereas the longer incubation time was included to ensure a readout from wells with few viable cells.

### Crystal violet staining

Evaluation of cell viability was performed using a crystal violet assay, according to a previously described protocol^27^. Cells were seeded in duplicate in 24-well plates and treated with the relevant compounds or vehicle control for 6 days. The medium and treatment compounds were replaced every 48 h. Experiments were repeated three times independently. After treatment, medium and unattached cells were removed, and the remaining cells were washed in phosphate-buffered saline (PBS) and fixed with glutaraldehyde for 15 min. The fixed cells were incubated with a crystal violet solution (0.1% in 20% methanol) for 1 h. The amount of dye taken up by the cells was determined by extracting the color with 10% acetic acid and measuring the absorbance at a wavelength of 595 nm. The maximal inhibitory concentration (IC_50_) was estimated by plotting the dose–response curve and determining the concentration at the point of 50% cell viability.

### Metabolic analysis

Metabolic analysis was performed using a Seahorse XFe96 Analyzer (Seahorse Bioscience, Billerica, MA). The Seahorse instrument performs real-time measurements of the extracellular acidification rate (ECAR) and oxygen consumption rate (OCR). Cells were seeded at a density of 20,000/well in Seahorse Cell Culture Microplates in RPMI1640 medium supplemented with 10% FBS one day prior to running the experiment. The test compounds were added 4 h after cell seeding and 24 h before the experiment. One hour before the experiment, the medium was replaced with Seahorse XF Base Medium supplemented with L-glutamine (2 mM) and pH adjusted to 7.4. The effects of the test compounds on the basal activities and capacities of the glycolytic and mitochondrial energy systems were determined by measuring ECAR and OCR during sequential injection of glucose (10 mM), oligomycin (2 µM), FCCP (1 µM) and rotenone/antimycin A (1/1 µM). All measurements were performed in sextuplicate. Basal measurements of OCR and ECAR were performed in medium without glucose, to determine the level of non-glycolytic ECAR. Glucose was injected into the medium, and the levels of OCR and ECAR were normalized to the basal rates. The ATP-synthase inhibitor oligomycin was then added to determine mitochondrial proton leakage, OCR-ATP coupling, and glycolytic capacity. To measure the maximal capacity of the mitochondrial respiration, the uncoupler FCCP was injected into the medium. The final step was the injection of rotenone and antimycin A, to inhibit all respiratory activity of the mitochondria and thereby reveal the level of non-mitochondrial oxygen consumption.

### Reactive oxygen species (ROS) evaluation

Thirty minutes before the end of the treatment, cells were incubated at 37 °C in serum-free DMEM with dihydroethidium (DHE, 5 µM), or with the mitochondrial superoxide specific probe MitoSOX (5 µM; Thermo-Fisher Scientific). Stained cells were washed twice with cold PBS, collected and analyzed by flow cytometry (FACS Verse, BD-Biosciences). For both probes, fluorescence intensity (geometric mean of the signal) was recorded (Ex/Em: 510/580 nm) and values were expressed as relative fold change with respect to vehicle-treated cells.

### Analysis of cell viability (Alamar Blue), apoptosis and cell cycle

Cell viability was determined at the end of the treatments by incubating the cells for 3 h with AlamarBlue Reagent (Thermo-Fisher Scientific), and following fluorescence emission at 590 nm. Values were expressed as relative fold change with respect to vehicle-treated cells.

Apoptotic cells and cell cycle profiles were evaluated after staining the cells with a solution containing 50 µM propidium iodide (Sigma-Aldrich), sodium citrate (1 mg/ml) and Triton-X100 (0.1% v/v). Fluorescence was recorded cytometrically using the FACS Verse. The percentage of apoptotic cells refers to the hypodiploid nuclei (SubG1) population. The percentage of cells in the different phases of the cell cycle was determined by identifying the propidium iodide fluorescence peaks corresponding to G1, S and G2 phases by FlowJo v 10.1 software.

### Immunoblotting

Immunoblotting was performed according to a previously described protocol^27^. Samples were prepared from cell culture flasks with lysis buffer (SLB) supplemented with colorless β-mercaptoethanol (BPB), Phospho-Stop and protease inhibitor (Thermo Fisher Scientific). Cell lysates were cleared by centrifugation 20,000 rpm for 3 min. Protein concentration was measured using the Qubit Protein Assay Kit (Thermo Fisher Scientific), and 50 µg protein of each sample were loaded to a 10-well SDS, 4–12% Bis–Tris NuPage gel (Invitrogen). Proteins were then separated at 80 V for 30 min, followed by 110 V until completion. Blotting was performed with a semi-dry transfer unit on an ECL nitrocellulose membrane at 3.3 mA/1 cm2/1 h/gel. Afterwards, the membrane was stained with Ponceau. The membrane was blocked in 5% milk for 1 h, then washed twice for 5 min with TBST and stained with AMPK or p-AMPK 1:2000 (Cell Signaling) in 5% BSA at 4 °C and with cyclophilin A 1:5000 (Cell Signaling) as loading control. After three 10-min cycles of washing with TBST, the membrane was stained with the secondary anti-body (Anti-Rabbit) 1:2000 for 1 h at room temperature, followed by another 3 wash cycles. Proteins were visualized using ECL Plus Western Blotting Substrate (Thermo Fisher Scientific) 1:1 for 2–3 min.

### Statistical analysis

For statistical analysis of variance between different treatments, one-way matched-samples ANOVA was used. Statistical significance was determined with Tukey’s honest significance difference (HSD) multi-comparison test.

## Supplementary Information

Supplementary Figures.

Supplementary Information.

Supplementary Table 1.
