# Supplementary material for: Screening of metabolic modulators identifies new strategies to target metabolic reprogramming in melanoma
Source: Sci Rep. 2021 Feb 23;11:4390. doi: 10.1038/s41598-021-83796-8 (PMC7902673; doi:10.1038/s41598-021-83796-8)
Supplement: Supplementary file 1 — Supplementary Figures. [file 41598_2021_83796_MOESM1_ESM.docx]

Supplementary Materials

**Screening of metabolic modulators identifies new strategies to target metabolic reprogramming in melanoma**

Cecilie Abildgaard^1,2^, Salvatore Rizza^3^, Helle Christiansen^4,5^, Steffen Schmidt^4,5^, Christina Dahl^1^, Ahmad Abdul-Al^1^, Annette Christensen^1^, Giuseppe Filomeni^3,6,7^, Per Guldberg^1,8^

^1^Molecular Diagnostics Group, Danish Cancer Society Research Center, Copenhagen, Denmark

^2^Department of Clinical Genetics, University Hospital of Southern Denmark

^3^Redox Signaling and Oxidative Stress Group, Danish Cancer Society Research Center, Copenhagen, Denmark.

^4^Lundbeckfonden Center of Excellence NanoCAN, Institute of Molecular Medicine, University of Southern Denmark, Odense, Denmark

^5^Molecular Oncology, Institute of Molecular Medicine, University of Southern Denmark, Odense, Denmark.

^6^Department of Biology, Tor Vergata University of Rome, Rome, Italy.

^7^Center for Healthy Aging, Copenhagen University, Copenhagen, Denmark

^8^Department of Cancer and Inflammation Research, Institute for Molecular Medicine, University of Southern Denmark, Denmark.

**Supplementary Fig. S1. Pipetting scheme.** The seeding protocol has 3 steps: 1) distribution of the library compounds into 96-well plates (final concentration 10 µM); 2) addition of either vemurafenib (final concentration 0.5 µM) or corresponding solvent (DMSO); and 3) addition of cells.


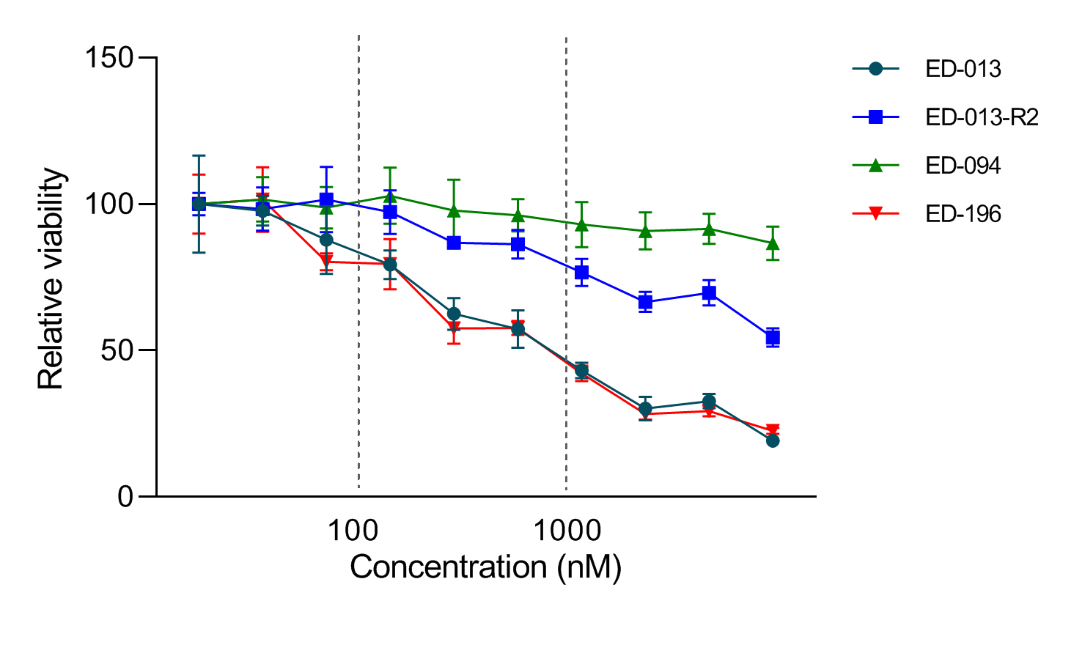


**Supplementary Fig. S2. Dose response to vemurafenib treatment.** Relative viability was determined with the CellTiter-Blue assay after 5 days of treatment with vemurafenib. Curves illustrate the response of sensitive (ED-013 and ED-196) and resistant (ED-094 and ED-013-R2) melanoma cells. Data points represent the mean of 6 replicates; error bars represent one SD from the mean.

**
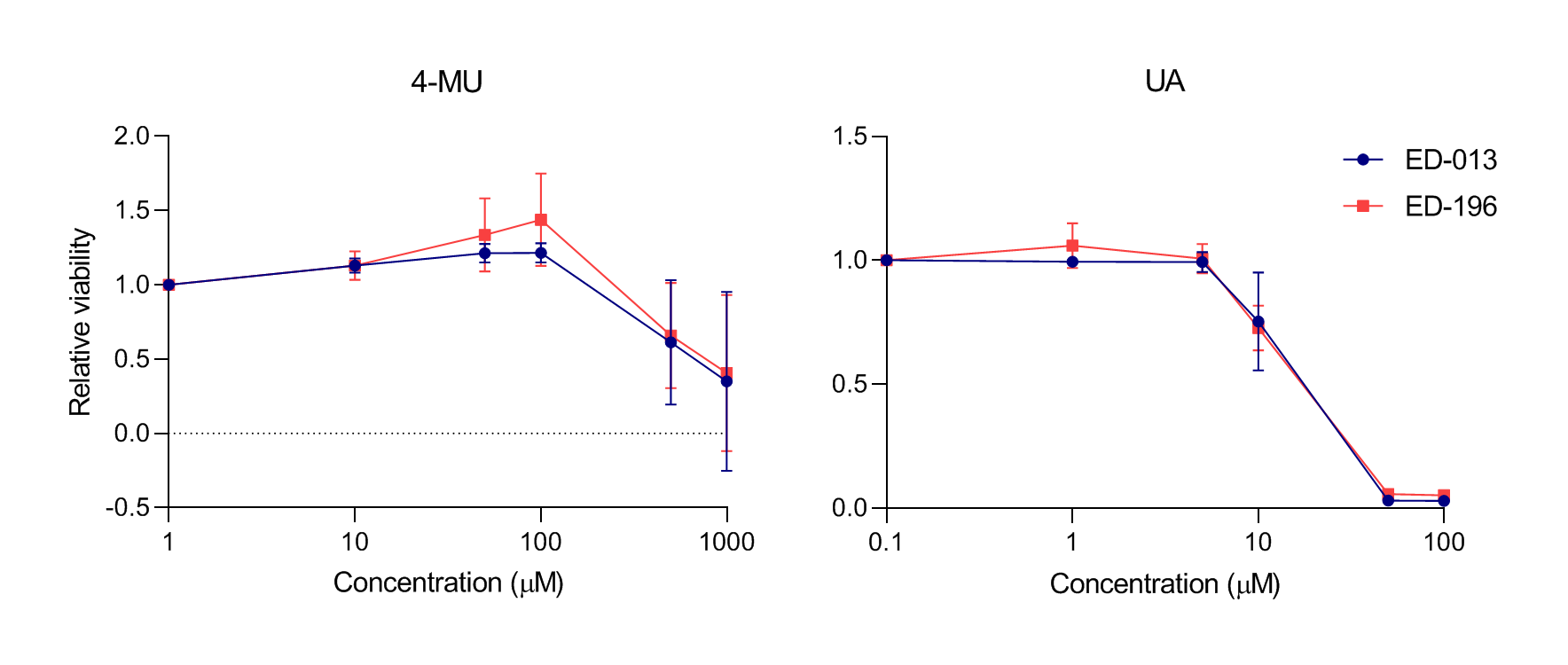
**

**Supplementary Fig. S3. Dose response to 4-MU and UA treatment.** Relative viability of ED-013 and ED-196 cells was determined with crystal violet staining after 6 days of treatment with 4-MU or UA. Data points represent the mean of ≥2 independent experiments; error bars represent one SD from the mean.

**
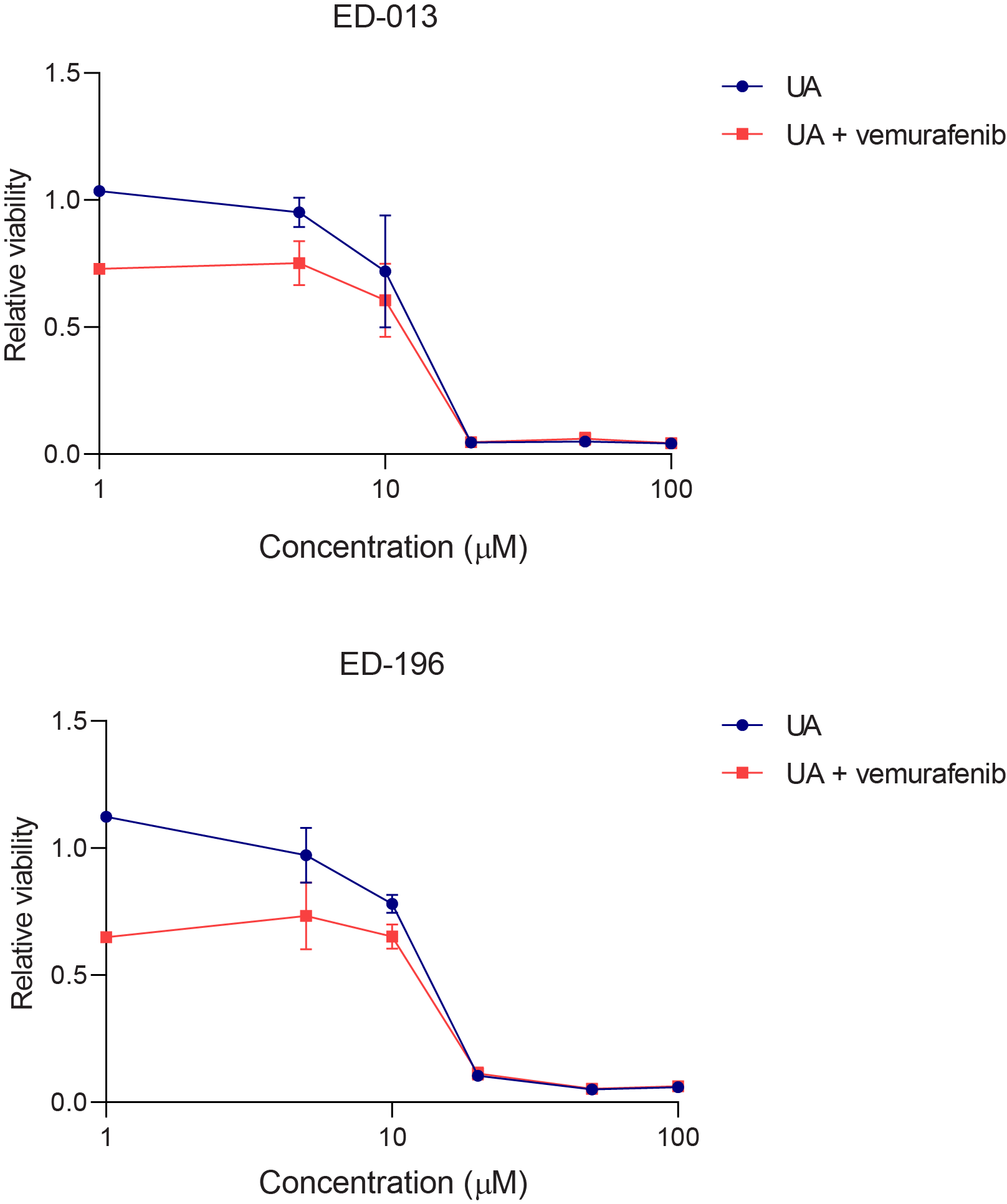
**

**Supplementary Fig. S4. Dose response to UA and the combination of UA and vemurafenib.** Relative viability of ED-013 and ED-196 cells was determined with crystal violet staining after 6 days of treatment with UA or UA + vemurafenib (0.1 μM). Repeated experiments were performed with different dose ranges also including other melanoma cell lines (data not shown). The dose-response curves shown are representative of our findings.

**
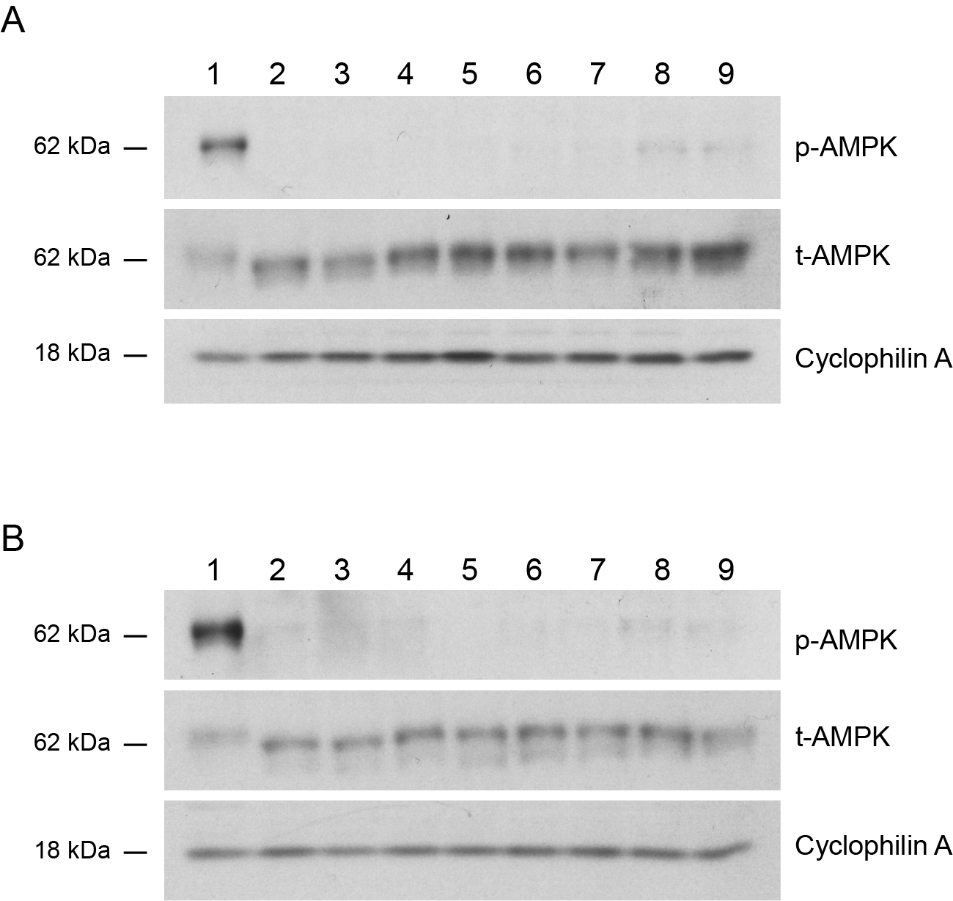
**

**Supplementary Fig. S5**. Immunoblot analysis. ED-196 melanoma cells were treated with a combination of UA (10 µM) and DCA (10 mM) (**A**) or a combination of 4-MU (400 µM) and vemurafenib (0.5 µM) (**B**) for 2 h (lane 3), 6 h (lane 5), 24 h (lane 7), or 48 h (lane 9). As control, cells were treated with DMSO alone for 2 h (lane 2), 6 h (Lane 4), 24 h (lane 6), or 48 h (lane 8). Cells treated with 5 µM LE-135 for 2 h served as positive control for AMPK phosphorylation (lane 1). Uncropped images of immunoblots are shown in Supplementary Fig. S6.


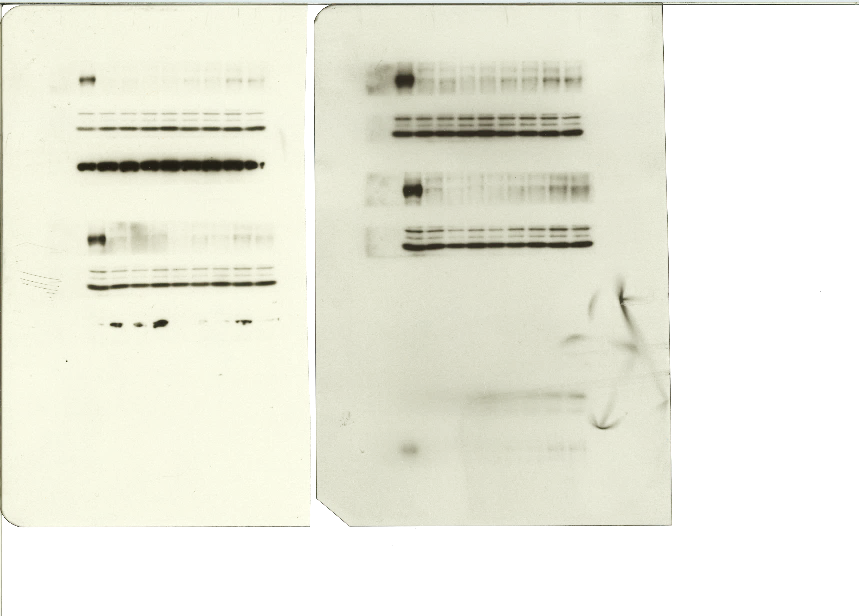

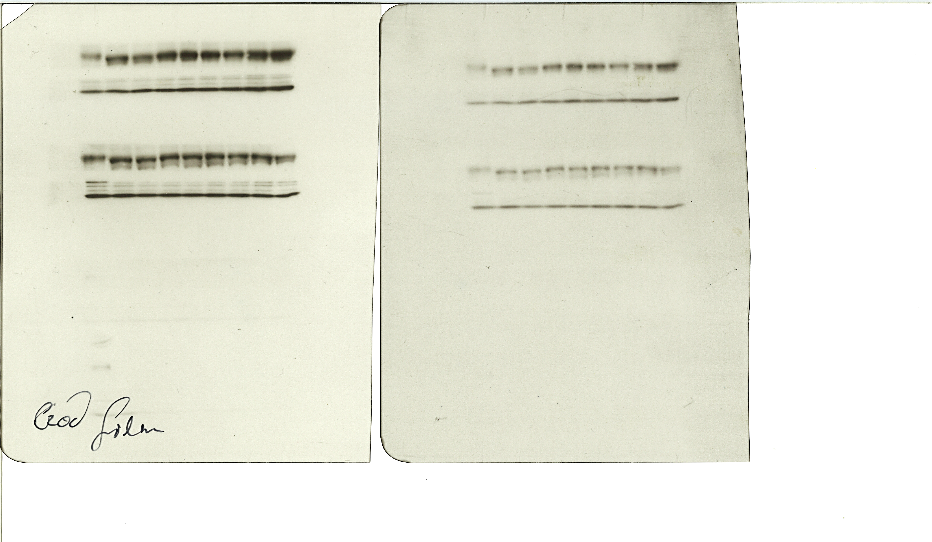


Low exposure

High exposure

High exposure

Low exposure

t-AMPK (panel A)

t-AMPK (panel B)

p-AMPK (panel A)

p-AMPK (panel B)

Cyclophilin A (panel A)

Cyclophilin A (panel B)

**Supplementary Fig. S6**. Uncropped images of immunoblots relating to Supplementary Fig. S5. Before incubation with antibodies, the nitrocellulose membranes were cut according to molecular weight.
