# Supplementary material for: Screening of metabolic modulators identifies new strategies to target metabolic reprogramming in melanoma
Source: Sci Rep. 2021 Feb 23;11:4390. doi: 10.1038/s41598-021-83796-8 (PMC7902673; doi:10.1038/s41598-021-83796-8)
Supplement: Supplementary file 2 — Supplementary Information. [file 41598_2021_83796_MOESM2_ESM.docx]

**Supplementary Table S1.** List of compounds ranked according to the sum of the differences between the relative viability of the metabolic modulators as single compounds and in combination with vemurafenib for both cell lines**.** The average of the differences between relative viability with and without vemurafenib plus the standard deviation is the selected cutoff. Positive hits are marked in red. Compounds marked in yellow were above the cutoff in both cell lines.
